# Supplementary material for: Myeloid GSK3α Deficiency Reduces Lesional Inflammation and Neovascularization during Atherosclerotic Progression
Source: Int J Mol Sci. 2024 Oct 10;25(20):10897. doi: 10.3390/ijms252010897 (PMC11507289; doi:10.3390/ijms252010897)
Supplement: Supplementary file 1 [file ijms-25-10897-s001.zip › ijms-3216222-supplementary.pdf]

International Journal of
Molecular Sciences

MDPI

Supplementary Materials:

Supplementary Table 1.

	Lαβfl/fl	LMαKO	LMβKO	LMαβKO
Body weight (g)	26.13±1.43	24.65±0.64	26.84±0.87	24.86±1.00
Liver weight (g)	1.34±0.12	1.20±0.09	1.32±0.08	0.91±0.12
Adipose weight (g)	0.42±0.07	0.28±0.06	0.53±0.07	0.49±0.24

Metabolic parameters of 15 weeks old LMαKO, LMβKO, LMαβKO and Lαβfl/ . Data are the mean ± SEM; n=7 mice/group.
